# Diagnosis of Gestational Diabetes Mellitus with Point-of-Care Methods for Glucose versus Hospital Laboratory Method Using Isotope Dilution Gas Chromatography-Mass Spectrometry as Reference

**DOI:** 10.1155/2020/7937403

**Published:** 2020-03-20

**Authors:** Karl Kristensen, Anne-Marie Wangel, Anastasia Katsarou, Nael Shaat, David Simmons, Helena Fadl, Kerstin Berntorp

**Affiliations:** ^1^Department of Obstetrics and Gynecology, Skåne University Hospital, Malmö, Sweden; ^2^Department of Clinical Sciences Lund, Lund University, Lund, Sweden; ^3^Faculty of Health and Society, Department of Care Science, Malmö University, Malmö, Sweden; ^4^Department of Endocrinology, Skåne University Hospital, Malmö, Sweden; ^5^Department of Clinical Sciences Malmö, Lund University, Lund, Sweden; ^6^Macarthur Clinical School, Western Sydney University, Campbelltown, Australia; ^7^Department of Obstetrics and Gynecology, Faculty of Medicine and Health, Örebro University, Örebro, Sweden

## Abstract

**Background:**

In Sweden, both glucose analyzers in accredited laboratories and point-of-care glucose devices are used for gestational diabetes mellitus (GDM) diagnosis. The aim of this study was to compare the diagnostic performance of the HemoCue Glucose 201+ (HC201+) and RT (HC201RT) systems with that of the hospital central laboratory hexokinase method (CL) based on lyophilized citrate tubes, using the isotope dilution gas chromatography-mass spectrometry (ID GC-MS) as reference.

**Methods:**

A 75 g oral glucose tolerance test was performed on 135 women screened positive for GDM. Diagnosis was based on the World Health Organization 2013 diagnostic thresholds for fasting (*n* = 135), 1 h (*n* = 135), 1 h (*n* = 135), 1 h (

**Results:**

Significantly more women were diagnosed with GDM by HC201+ (80%) and CL (80%) than with the reference (65%, *P* < 0.001) based on fasting and/or 2 h thresholds, whereas the percentage diagnosed by HC201RT (60%) did not differ significantly from the reference. In Bland-Altman analysis, a positive bias was observed for HC201+ (4.2%) and CL (6.1%) and a negative bias for HC201RT (−1.8%). In the surveillance error grid, 95.9% of the HC201+ values were in the no-risk zone as compared to 98.1% for HC201RT and 97.5% for CL.

**Conclusions:**

A substantial positive bias was found for CL measurements resulting in overdiagnosis of GDM. Our findings suggest better performance of HC201RT than HC201+ in GDM diagnosis. The results may have possible implications for GDM diagnosis in Sweden and require further elucidation.

## 1. Introduction

The oral glucose tolerance test (OGTT) is the gold standard for diagnosis of gestational diabetes mellitus (GDM). In 2015, the Swedish National Board of Health and Welfare (SNBHW) reviewed the evidence of the current Swedish and the World Health Organization (WHO) diagnostic criteria for GDM and recommended a shift to the lower WHO diagnostic thresholds [1]. These are based on the thresholds from the Hyperglycemia and Adverse Pregnancy Outcome (HAPO) study that identified pregnancies in which the risks of various adverse outcomes were increased, with an adjusted odds ratio of 1.75 compared to mean glucose concentrations [2, 3]. Due to the limited evidence base, the SNBHW made no recommendations regarding screening or method of glucose analysis. New in the guidelines was the explicit limitation of glucose measurements in venous blood samples. GDM screening strategies and diagnostic approaches have varied in Sweden over the years [4]. Thresholds have been based on either venous or capillary blood sampling, and for glucose analysis, either hospital laboratory methods or point-of-care (POC) glucose instruments have been used. The HemoCue Glucose 201+ (HC201+) and HemoCue Glucose 201 RT (HC201RT) systems (HemoCue, Ängelholm, Sweden) are examples of such methodology and are widely used in Sweden for GDM diagnostic purposes.

There has been doubt among healthcare professionals in Sweden as to whether to implement the new lower thresholds, as the number of women expected to be diagnosed with GDM would increase considerably [5], raising concerns over the clinical management and the associated economic costs. To address this reluctance, members of the former expert group appointed by the SNBHW to review the current GDM guidelines agreed to facilitate the transition to new criteria by means of a trial. Eventually, a stepped-wedge randomized controlled trial was designed, the CDC4G (Changing Diagnostic Criteria for the Diagnosis of Gestational Diabetes) trial (study ID ISRCTN41918550, 15/12/2017), with the aim to evaluate the clinical and health economic impacts of changing diagnostic criteria for GDM in Sweden [6]. The results of the trial are currently under evaluation and will be presented elsewhere.

Recruitment to CDC4G took place during 2018. By this time, all participating centers had changed to venous blood sampling during the diagnostic OGTT but otherwise continued their usual screening approach. The antenatal center in Malmö was one of 11 centers that agreed to participate. In Malmö, blood samples obtained during the diagnostic OGTT were analyzed by the hospital laboratory method. In parallel, glucose was measured by HC201+ to give a preliminary immediate result to the women. In the introductory months of the recruitment, it was observed that samples analyzed by the hospital laboratory method resulted in somewhat higher glucose concentrations than those analyzed by HC201+. This prompted us to undertake a substudy comparing the diagnostic value of the HemoCue Glucose 201 systems and the current hospital laboratory method for glucose measurement, with that obtained by a gold standard technique, i.e., the isotope dilution gas chromatography-mass spectrometry (ID GC-MS).

## 2. Material and Methods

### 2.1. Study Population

In Malmö, screening for GDM with a 75 g OGTT is offered to all women in the twenty-eighth week of gestation at their local antenatal clinic, and also in gestational week 12 if they have a history of GDM or macrosomia in previous pregnancies, a first-degree relative with diabetes, or body mass index (BMI) ≥ 35 kg/m^2^. The diagnostic criteria for GDM are a slight modification of those recommended by the European Association for the Study of Diabetes defining GDM as a fasting capillary plasma glucose concentration of ≥ 7.0 mmol/L and/or a 2 h capillary plasma glucose concentration of ≥ 10.0 mmol/L [7]. The HemoCue Glucose 201 systems are used to perform immediate analysis of blood glucose concentrations. To ascertain the quality of the individual testing, double sampling is used with acceptance of a divergence of ≤ 0.3 mmol/L. In the clinical setting, the highest test result is regarded as the diagnostic value. During the recruitment period to the CDC4G study, women who screened positive for GDM at their local antenatal clinic in the catchment area of Malmö were referred to the specialist antenatal clinic at the hospital for a diagnostic OGTT. All women referred from the beginning of March to the end of December 2018 were eligible for inclusion in the present study. Altogether, 135 out of 149 eligible women underwent an OGTT according to the study protocol as outlined below. Reasons for nonparticipation was refusal (*n* = 4), nonfasting state (*n* = 3), impossible to insert a venous cannula (*n* = 6), and miscarriage (*n* = 1). Between March and August 2018, glucose concentrations were determined at fasting and 2 h after the glucose load, and after randomization to switching to the WHO 2013 criteria (September–December 2018), a 1 h sample was added according to the protocol of the stepped-wedged study design. For the purpose of the present study, we used the following thresholds to define GDM for the whole study period: fasting glucose ≥ 5.1 mmol/L, and/or 1 h glucose ≥ 10.0 mmol/L (if taken), and/or 2 h glucose ≥ 8.5 mmol/L.

The study protocol was approved by the Ethics Committee of Uppsala University (2016/487/2). All the women who were included received written information about the study and signed a written informed consent document.

### 2.2. Study Procedure and Blood Collection

A standard 75 g OGTT was performed in the morning after overnight fasting, by either one of two specially trained midwives. A venous cannula was inserted into an antecubital vein. At the respective time point of the OGTT, three blood samples were taken for determination of glucose concentrations as described below.

#### 2.2.1. ID GC-MS

The first sample was collected into a lithium heparin tube with gel and immediately centrifuged at 2,000 g for 10 min (Sigma 2-6 E Laboratory Centrifuge). After centrifugation, the plasma was transferred into a microtube without additives and stored at −20°C until analysis. This sample was used as the reference standard for the study. All samples were labeled with a code number, and analyses were performed blind.

*(1) Apparatus*. The instrument used for GC-MS analysis was a Shimadzu GC-2010 Plus gas chromatograph with a Shimadzu AOC-20s autosampler and a Shimadzu AOC-20i autoinjector. The detection was done with a Shimadzu QP2020 EI single quadrupole detector. The separation was done with a 30 m × 0.25 mm Rxi-5ms fused-silica column coated with a 0.25 *μ*m film (Restek Corporation, Bellefonte, PA). The carrier gas was helium. The injector was in split mode, and the injector temperature was kept at 270°C. The oven temperature gradient was ramped from 80°C to 300°C at 40°C/min. The derivatives of glucose and [^13^C_6_]-glucose eluted after ~5.9–6.1 min. The column transfer line to the ion source was kept at 260°C. Selected ion monitoring was carried out with acquisition of ions at *m*/*z* 314.0 and 242.1 for unlabeled glucose and acquisition of ions at *m*/*z* 319.0 and 246.1 for [^13^C_6_]-labeled glucose.

*(2) Standard Solutions and Controls*. Standard solutions were prepared from glucose Standard Reference Material 917c (D-Glucose; NIST, Gaithersburg, MD). The standard solutions were prepared by weighing the reference material with an AX105 Delta Range analytical balance (Mettler Toledo) and dilution with deionized water using volumetric pipettes and volumetric flasks to six different concentrations: 1, 3, 6, 10, 20, and 30 mmol/L. To assess the accuracy of the method, Standard Reference Material 965b (glucose in frozen human serum; NIST) Level 1 to Level 4 was used [8]. The defined value at each level is 33.08 mg/dL (1.83 mmol/L), 75.56 mg/dL (4.20 mmol/L), 118.50 mg/dL (6.58 mmol/L), and 294.50 mg/dL (16.36 mmol/L), respectively. The averages for each level from the thirteen occasions deviated by −1.8%, −0.5%, −1.1%, and −0.3%, respectively, from the defined values.

*(3) Internal Standard Solution*. Internal standard solution was prepared by weighing [^13^C_6_]-glucose (Cambridge Isotope Laboratories, Andover, MA) with an AX105 Delta Range analytical balance (Mettler Toledo) and dilution with deionized water using volumetric pipettes and volumetric flasks, to a final concentration of 1.0 mmol/L.

*(4) Chemicals*. All chemicals were of analytical grade. Hydroxylamine hydrochloride and pyridine were from Sigma-Aldrich. Ethanol was from Acros Organics. Methanol was from Fluka and Honeywell. Chloroform was from Honeywell.

*(5) Sampling Equipment and Analytical Procedure*. The analyses were distributed over 11 months on thirteen different occasions between March 27, 2018, and January 24, 2019. The ID GC-MS method used was performed essentially as previously described by Hannestad and Lundblad for glucose in whole blood [9]. The samples were thawed on a roller mixer at room temperature. The plasma was then transferred to Eppendorf tubes and centrifuged at 2,000 g for 10 min (Sigma 1-14 Laboratory Centrifuge). This step was introduced to prevent clots from affecting further analysis. The plasma sample (40 *μ*L) and the internal standard solution (160 *μ*L) were collected with a Microlab 540B diluter/dispenser (Hamilton Company, Reno, NV). Plasma samples were diluted with internal standard solution and transferred to blood collection tubes (BD Vacutainer, Z, 5 mL). The tubes were shaken thoroughly. The samples were left on the bench at room temperature for 1–2 h, and 0.8 mL of ethanol/methanol solution (50/50) was added to them for deproteinization. The tubes were shaken thoroughly. The samples were then left on the bench for 30–45 min, followed by centrifugation for 10 min at 2,000 g (Eppendorf Centrifuge 5416). A proportion of the upper phase (0.5 mL) was transferred to a derivatization tube (16 × 55 mm, with screw cap) and dried without the cap, under a stream of air on a heating block (Techne Sample Concentrator, Dri-Block 3D) at 50°C for 30 min. Hydroxylamine hydrochloride solution (150 *μ*L, 0.2 M) in pyridine was added to the tube, and the glucose was converted to aldonitrile derivatives by heating at 90°C for 40 min (Heraeus VT 5042 EK heating cabinet). After cooling, the derivatives were acetylated by addition of 200 *μ*L acetic anhydride, followed by heating at 90°C for 60 min. The samples were then evaporated to dryness in air at 50°C, and the residues were dissolved in 0.5 mL chloroform. One hundred fifty *μ*L of the solution was diluted with 1,350 *μ*L chloroform in vials (1.5 mL GC vials). The vials were capped and loaded into the autosampler. Standard solutions and controls were diluted and analyzed in the same way as the plasma samples. The injector was programmed to inject 1 *μ*L of each sample into the gas chromatograph. All samples, standards, and controls were measured in duplicate, and the results were quantified by measuring the peak areas for *m*/*z* 314.0 for unlabeled glucose and *m*/*z* 319.0 for [^13^C_6_]-labeled glucose. The *m*/*z* 242.1 and 246.1 ions were used as qualifier ions and served to confirm the identity of the glucose and [^13^C_6_]-labeled glucose derivatives. Calibration curves were produced, and the concentration of glucose in unknown samples was calculated from a linear regression fit of the peak area ratios (*m*/*z* 314.0)/(*m*/*z* 319.0). The results were noted as the average of the duplicates.

#### 2.2.2. HemoCue Glucose 201

The second sample was collected into an EDTA tube. From this sample, a small amount of blood was placed on the inside of the respective cuvette folder for simultaneous and immediate analysis with HC201+ and HC201RT. The procedure was repeated for a duplicate test. All calculations were performed on mean values from the duplicates. Mean imprecision (CV%) of the duplicate samples was 2.5 for HC201+ and 2.9 for HC201RT. The HemoCue systems use a photometric technique for glucose analysis that is traceable to ID GC-MS [9]. The instrument automatically converts blood glucose concentrations to plasma glucose concentrations using a conversion factor of 1.11 [10]. The major difference between the two systems is that the 201+ cuvettes must be kept at 4–8°C whereas the 201RT cuvettes can be kept at room temperature and therefore have a different design, which excludes the possibility of prereaction at room temperature. Two different lot numbers of HC201+ and HC201RT cuvettes were used in the study, with a switch during the summer. The instruments were checked every week with a calibration fluid supplied by the company (Gluco Trol-NG). A log was kept and there were no discrepancies. Participation in external quality assessment (EQA) schemes from Equalis (a provider of EQA for clinical laboratory investigations in Sweden) took place on a monthly basis. The median (range) percentage deviation from the assigned glucose values (6.8–15.0 mmol/L) was 1.2 (–2.1 to +4.4) for HC201+ and 2.0 (–1.7 to +8.1) for HC201RT.

#### 2.2.3. Hospital Central Laboratory Hexokinase Method

The third sample was collected into a VACUETTE® FC Mix Tube (Greiner Bio-One, containing a lyophilized mixture of Na2EDTA, sodium fluoride, citric acid, and sodium citrate) and was kept at room temperature until analysis within 3–4 h. The midwife delivered the samples to the laboratory directly after completion of the OGTT. Glucose was analyzed on a Cobas 8000 modular analyzer (Roche Diagnostics Ltd., Rotkreuz, Switzerland) by a hexokinase method (hereon referred to as CL) traceable to the glucose ID GC-MS. The method has shown a mean imprecision (CV%) of 1.0 and 1.1 at plasma glucose levels 3 mmol/L and 20 mmol/L, respectively (*n* = 50). The laboratory is accredited according to SS-EN ISO 15189 and participates in interlaboratory comparison schemes from Equalis (https://www.equalis.se/en/).

All women had their BMI calculated at their first antenatal visit. Clinical and sociodemographic details were recorded at the time of the diagnostic OGTT at the hospital.

### 2.3. Statistical Analysis

All comparisons were performed with ID GC-MS measurements as the reference. Normality was assessed visually and by the Shapiro-Wilk test. Differences in mean glucose concentrations were determined using the paired Student *t*-test. The impact of the different methods of glucose analysis on the proportions of women diagnosed with GDM was determined by the McNemar test for correlated proportions. Bonferroni correction was used to adjust for repeated comparisons. Pearson's test was used for estimation of correlations.

Modified Bland-Altman analysis was used to compare the differences between glucose values obtained by each method and the reference method with glucose concentrations obtained by the reference method. Surveillance error grid analysis was performed for each method of glucose analysis and was compared with the reference measurements according to the methodology described by Klonoff et al. [11], using the surveillance error grid software copyrighted by the University of Virginia (Charlottesville, VA) and available through the Diabetes Technology Society website at http://www.diabetestechnology.org/SEGsoftware [12]. The surveillance error grid displays clinical risks on a continuous color-coded scale relevant to clinical practice as perceived by diabetes experts.

The International Organization for Standardization (ISO) has been used by regulatory agencies to determine whether a blood glucose meter is sufficiently accurate to be marketed commercially for the use of people with diabetes. The current ISO 15197: 2013 accuracy criteria require that ≥95% of all meter results fall within ±0.83 mmol/L (±15 mg/dL) or ±15% of the reference results at glucose concentrations of <5.55 mmol/L (<100 mg/dL) or ≥5.55 mmol/L (≥199 mg/dL), respectively [13]. Comparison of the surveillance error grid with ISO 15197: 2013 using computer-simulated pairs with realistic error distribution suggests that a device with ≤3% errors outside the surveillance error grid no-risk “green” zone would meet the ISO requirements of ≤5% data pairs outside the 0.83 mmol/L or 15% standard limits, while higher percentages outside the surveillance error grid no-risk zone would indicate noncompliance with the standard [12].

IBM SPSS Statistics 24 for Windows (IBM Corporation, Armonk, NY) was used for analysis. Two-sided *P* values less than 0.05 were considered to be statistically significant.

## 3. Results

The mean age of the 135 women studied was 32.6 ± SD 5.4 years; the mean BMI was 26.9 ± 5.2 kg/m^2^; the median gestational age was 30.1 (range 12.3–37.3) weeks; the median hemoglobin concentration was 116.9 (93–144) g/L; 33.3% were nulliparous; 5.2% smoked; 44.4% were of European origin (half of them Swedish); and 55.6% were of non-European origin (with Arab and Asian origin being the largest groups).

Table 1 shows a comparison of the mean glucose results for HemoCue and CL measurements with those obtained by the reference method (ID GC-MS). HC201+ resulted in higher mean glucose concentrations than those obtained by HC201RT (*P* < 0.001), with a similar trend before and after the switch of lot numbers during the summer (data not shown).

Table 2 is a comparison of the number of abnormal results for the fasting, 1 h, and 2 h samples for HemoCue and CL measurements with the number of abnormal results from the reference method. The best diagnostic performance relative to the reference was found for HC201RT.

Glucose concentrations at fasting, 1 h, and 2 h correlated significantly with the reference method (*P* < 0.001) for all the measurements: *r* = 0.87, 0.95, and 0.97, respectively (HC201+); *r* = 0.87, 0.96, and 0.97, respectively (HC201RT); and *r* = 0.96, 0.97, and 0.98, respectively (CL).

**Figure 1:**
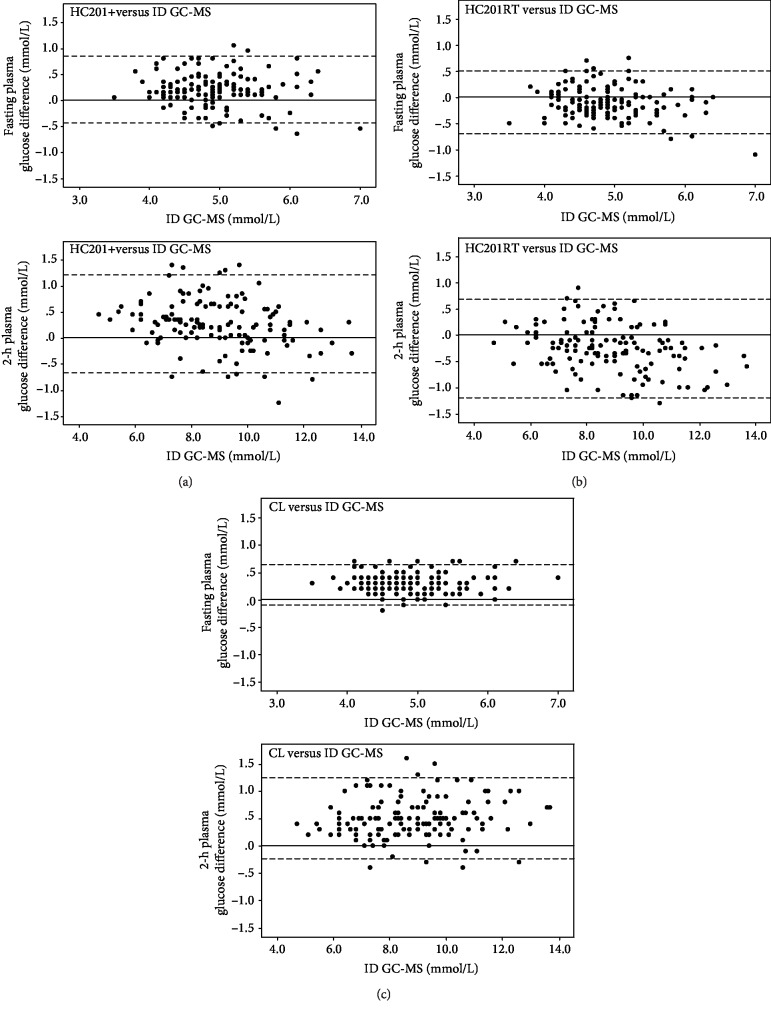
Modified Bland-Altman plots of fasting and 2 h glucose measurements for HC201+ (a), HC201RT (b), and CL (c). The dashed lines show the range containing the mean of the differences ± 1.96 SD. CL: central laboratory; HC201+: HemoCue 201+; HC201RT: HemoCue 201 RT; ID GC-MS: isotope dilution gas chromatography-mass spectrometry.

**Figure 2:**
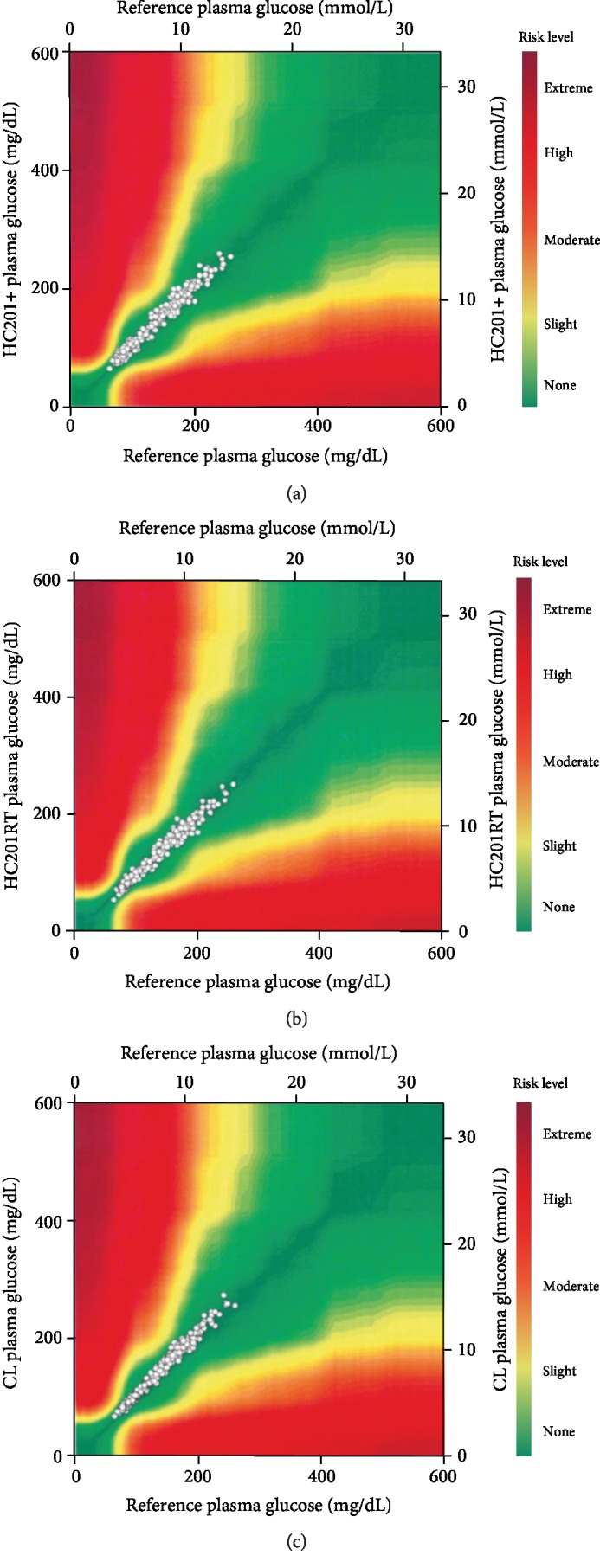
Surveillance error grid analysis for HC201+ (a), HC201RT (b), and CL (b) relative to the reference method of glucose measurement. The color-coded risk zone definition is according to Klonoff et al. [11]. CL: central laboratory; HC201+: HemoCue 201+; HC201RT: HemoCue 201 RT.

Modified Bland-Altman plots for fasting and 2 h glucose measurements for the different methods of glucose analysis are shown in Figure 1.  Table 3 is a summary of the overall results of the modified Bland-Altman analysis, comparing the difference (glucose value obtained by each method minus the reference value) with the reference value. All three methods met the ISO 15197: 2013 accuracy standard of ≥95% compliant pairs [13]. The numbers of compliant pairs were 309 (97.5%) for HC201+, 315 (99.4%) for HC201RT, and 309 (97.5%) for CL. However, in the surveillance error grid analysis (Figure 2), 95.9% of the HC201+ values were within the no-risk (deep green) zone, as compared to 98.1% of the HC201RT values and 97.2% of the CL values. The remaining values were within the slight-risk zone (light green), indicating noncompliance with the ISO standard for HC201+ as predicted by Kovatchev et al. [12].

There were no significant correlations between hemoglobin concentrations and glucose concentrations obtained by either one of the two HemoCue systems at any time point of the OGTT (data not shown).

## 4. Discussion

Using the WHO 2013 thresholds for the diagnosis of GDM, we found that HC201RT was more accurate than both HC201+ and CL glucose measurements in making the diagnosis of GDM in women screened positive with a universal 75 g OGTT based on capillary blood sampling and modified EASD criteria [7]. Furthermore, in modified Bland-Altman analysis, HC201RT showed the best agreement with the reference method. CL measurements consistently yielded higher glucose values than the reference method, resulting in a substantial bias in the Bland-Altman analysis and an increased number of women diagnosed with GDM. The findings may have possible implications for the clinical setting of GDM diagnosis in Sweden.

Optimal conditions for glucose measurements are crucial in GDM diagnosis. Unsatisfactory glycolysis inhibition may lead to diagnostic misclassification. Thus, a key component of the HAPO study was the standardization of research conditions with particular attention being paid to sample handling and centralization of glucose measurements [14]. However, in 2011, the laboratory standards applied in the HAPO study were revised. To inhibit glycolysis more effectively, it was recommended that the fluoride sample tube (kept on ice) should be centrifuged within 30 min and not within 60 min as previously recommended [15]. If this is not possible, tubes containing a rapidly effective glycolysis inhibitor, such as citrate buffer, should be used [15]. The acidification of whole blood with citrate buffer seems to stop glycolysis immediately [16]. Gambino et al. showed that the mean glucose concentrations decreased by 0.3% and 1.2%, respectively, 2 h and 24 h after collection at room temperature [17]. At present, most accredited laboratories in Sweden have changed to tubes with lyophilized citrate. Due to the very effective inhibition of glycolysis, this change may lead to a more accurate estimation of plasma glucose concentrations and so to overdiagnosis of GDM if diagnostic thresholds are based on the HAPO study where less efficient inhibition of glycolysis was used [18, 19]. This highlights the importance of the ongoing CDC4G trial to define diagnostic cut-off points for the diagnosis of GDM in a contemporary Swedish setting.

Not surprisingly, we found a strong correlation between CL measurements and those obtained using the reference method. Likewise, the Bland-Altman plots indicated a high precision of the method. However, the finding of an overall positive bias of 6.1% for CL measurements, most pronounced for 1 h measurements, was more than expected. The calculated bias for CL measurements at the different time points of the OGTT (Table 3) correspond to fasting, 1 h, and 2 h values of 5.4 mmol/L, 10.8 mmol/L, and 9.0 mmol/L at diagnostic cut-off points, respectively, leading to a proportional increase in GDM cases (Table 2). Other groups have reported a small positive bias for glucose concentrations measured in citrate buffer tubes when compared to glucose values measured in conventional tubes under optimal preanalytical conditions [16, 20, 21]. However, still other groups have reported good agreement in glucose results between the different types of collecting systems [22–24]. A suboptimal inhibition of glycolysis in the reference tube may account for some of these differences, considering that glycolysis takes place within minutes from venipuncture until plasma is separated [20]. For the present study, the reference tube was centrifuged immediately with no delay, but it cannot be excluded that some glycolysis did take place, as we did not have access to chilled centrifugation. Inaccuracy of the reference method should also be considered to be a potential source of bias. However, only minor deviations from reference values were found when the accuracy was assessed in certified values (NIST) at four levels.

To comply with international guidelines [15], although not mandatory, eight of the eleven centers participating in the CDC4G trial used accredited laboratory methods for glucose measurements during the study period [6]. All methods (Roche Cobas, Beckman Coulter Au, Siemens Advia, or Abbott Architect) were based on hexokinase [6]. The samples were drawn in EDTA tubes with citrate and sodium fluoride as an antiglycolytic agent. During the study period (2018), the mean bias in relation to results from reference measurement procedures in EQA was 3.7% for all the participating hospital laboratories in CDC4G and 3.2% for the hospital laboratory in Malmö (Equalis, Uppsala). The reasons for this positive bias is unclear, but may possibly relate to a remaining inaccurate calibration of instruments by the manufacturers as once pointed out by Gambino [25], and consistent with a report by Miller et al. [26].

To our knowledge, this is the first study to evaluate lyophilized citrate tubes for the diagnosis of GDM in a clinical setting, in a rather large number of women at a high risk of GDM using WHO 2013 diagnostic thresholds. Previous studies have shown no or only a small increase in the diagnoses of GDM with the use of lyophilized citrate tubes [22, 24]. However, these studies were based on smaller numbers of women with generally lower glucose levels and few patients close to the diagnostic cut-off levels. A mean difference of 0.3 mmol/L fasting and up to 0.8 mmol/L postload, as noted in the present study, will lead to overdiagnosis of GDM in the large proportion of women who have glucose concentrations close to the diagnostic cut-off points.

Glucose measurement by a POC technique for the diagnosis of GDM is useful for both the women and their caregivers. This saves time, reduces costs, and reduces some of the preanalytical errors. Furthermore, it has the advantage of providing a diagnosis immediately so that treatment can start promptly. In previous comparisons with laboratory glucose measurements, the HemoCue Glucose 201 system has been found to be accurate [27, 28]. When HC210RT was compared to accredited hospital laboratory methods in subjects with coronary artery disease in a European survey, the Bland-Altman plot showed small differences and, in contrast to our findings, a tendency of higher glucose measurements for HC201RT than for hospital laboratory measurements [28]. The −1.8% bias noted for the HC201RT instrument in the present study is close to the 2.2% limit for acceptable bias according to international guidelines [15]. However, HC201+ performed less well than HC201RT in Bland-Altman and surveillance error grid analysis, and plasma glucose recordings were consistently higher than those by HC201RT were. This contrasts with a previous study showing only minor deviations between the two devices [27]. We postulated that variations between different lot numbers of cuvettes might have a role in these results. However, similar deviations in glucose values between the two devices were found before and after the change in lot numbers during the summer. Moreover, good agreement between different HemoCue devices of the same model and lot numbers has been demonstrated previously [29]. According to the manufacturer, it can be expected for both devices to have a lot-to-lot as well as an instrument-to-instrument variation of less than 0.2 mmol/L or a total system variation of less than 0.3 mmol/L at normal glucose levels. In the present study, the deviations in mean values between the two devices were most pronounced for 1 h and 2 h values, which may indicate a larger system variation at higher glucose levels. Furthermore, although we followed the manufacturer's instructions for storage of the HemoCue201+ cuvettes at 4–8°C, we cannot exclude the possibility that this potential source of error may have affected the results.

The constant factor 1.11 used by the HemoCue system for automatic conversion of whole blood glucose values to equivalent plasma glucose values is based on the relationship at normal hematocrit (0.43) [10]. Hematocrit levels at the extremes will therefore have an effect on the results [29]. Considering the relative anemia that occurs in pregnancy due to hemodilution, the conversion factor 1.11 may not be optimal [30]. Using hemoglobin concentrations (range 93–144 g/L) as a surrogate measure of hematocrit, we found no correlation with the HemoCue glucose results in the present study group. However, this does not preclude an effect at the individual level. A bias of 5% could be expected at the hematocrit extremes 0.25 and 0.6, and a conversion factor of 1.06 and 1.16, respectively, would have been more appropriate to use under these circumstances [10].

A major strength of this study was the access to the ID GC-MS technology as a reference method. Furthermore, two specially trained midwives conducted the study under standardized conditions in a clinical setting. A possible limitation of this study was that we did not undertake experiments to explore the reasons for the high bias of CL measurements, such as head-to-head comparisons with ID GS-MS in samples collected and handled under identical conditions. Unfortunately, the reference method was not included in the EQA schemes from Equalis. Owing to the fact that the study was confined to a single center in the CDC4G trial, the results cannot automatically be extrapolated to other regions of Sweden using other models for GDM screening and diagnostic procedures.

## 5. Conclusions

Our results showed substantial positive bias from the hospital laboratory procedure for glucose measurements resulting in overdiagnosis of GDM. Furthermore, HC201RT showed better agreement with the reference than HC201+. The HemoCue Glucose 201 systems are widely used in Sweden for GDM screening and diagnosis. Our findings support the continuous use of HC201RT in GDM diagnostics. However, more work on the HemoCue systems across hematocrit levels in pregnancy is desirable. Finally, there is an urgent need to explain the reasons for the high bias of the CL glucose measurements and to confirm our results in different settings of GDM diagnosis in Sweden.

## Figures and Tables

**Table 1:** Comparison of mean glucose concentrations (mmol/L) between each method and the reference method.

	Fasting (*n* = 135)	1 h (*n* = 52)	2 h (*n* = 135)
ID GC-MS	4.9 ± 0.6	10.0 ± 1.7	8.9 ± 1.8
HC201+	5.1±0.5^∗∗∗^	10.5±1.7^∗∗∗^	9.1±1.8^∗∗∗^
HC201RT	4.8±0.6^∗∗^	10.1 ± 1.6	8.6±1.7^∗∗∗^
CL	5.2±0.6^∗∗∗^	10.8±1.8^∗∗∗^	9.4±1.9^∗∗∗^

Data are mean ± SD. ^∗∗^*P* < 0.01 and ^∗∗∗^*P* < 0.001 compared to ID GC-MS (paired Student *t*-test using Bonferroni correction). CL: central laboratory; HC201+: HemoCue 201+; HC201RT: HemoCue 201 RT; ID GC-MS: isotope dilution gas chromatography-mass spectrometry.

**Table 2:** Comparison of abnormal test results between each method and the reference method.

	Fasting (*n* = 135)	2 h (*n* = 135)	Fasting and/or 2 h (*n* = 135)	1 h (*n* = 52)	Fasting and/or 1 h (*n* = 52)	Overall^a^ (*n* = 52)
ID GC-MS	45 (33)	71 (54)	86 (65)	24 (47)	28 (55)	35 (70)
HC201+	64 (47)^∗∗∗^	89 (66)^∗∗∗^	108 (80)^∗∗∗^	34 (67)^∗^	36 (71)	44 (85)
HC201RT	41 (30)	69 (51)	82 (61)	22 (43)	27 (53)	35 (67)
CL	75 (56)^∗∗∗^	86 (64)^∗∗∗^	107 (80)^∗∗∗^	38 (73)^∗∗∗^	42 (81)^∗∗∗^	45 (87)^∗^

Data are *n* (%). Fasting cut-off of ≥5.1 mmol/L; 1 h cut-off of ≥10 mmol/L; 2 h cut-off of ≥8.5 mmol/L. ^a^Fasting and/or 1 h and/or 2 h. ^∗^*P* < 0.05, ^∗∗^*P* < 0.01, and ^∗∗∗^*P* < 0.001 compared to ID GC-MS (McNemar test for correlated proportions using Bonferroni correction). Missing values were below 4% for all measurements. CL: central laboratory; HC201+: HemoCue 201+; HC201RT: HemoCue 201 RT; ID GC-MS: isotope dilution gas chromatography-mass spectrometry.

**Table 3:** Summary of modified Bland-Altman comparison.

	Valid samples (*n*)	Bias (%)	MARD (%)^a^	CV (%)^b^	95% limits of agreement	
					Lower^c^	Upper^d^
HC201+						
Fasting	135	4.3	6.3	6.5	–7.6	16.5
1 h	50	4.9	5.8	5.2	–3.7	14.6
2 h	132	3.5	5.1	5.6	–6.7	13.4
Overall	317	4.2	5.8	6.0	−7.6	15.9
HC201RT						
Fasting	135	–1.8	5.1	6.1	–12.2	9.4
1 h	50	0.3	3.4	4.3	–6.5	8.3
2 h	132	–2.7	4.8	5.2	–12.1	6.7
Overall	317	−1.8	4.7	5.6	−12.8	9.2
CL						
Fasting	134	5.9	6.0	3.8	0.0	13.0
1 h	51	8.1	8.2	4.3	0.7	14.8
2 h	132	5.7	6.0	4.3	–0.9	15.1
Overall	317	6.1	6.3	4.2	−2.1	14.3

Modified Bland-Altman analysis comparing the difference (glucose value obtained by each method–reference value) with the reference value. The bias is the mean relative difference as a percentage of the reference value. A bias of 5% means that the value is on average 5% higher than the reference value. ^a^Mean absolute relative difference as a percentage of the reference value. ^b^Standard deviation of the bias. ^c^Lower 95% limits of agreement define bias −1.96 ∗ CV. ^d^Upper 95% limits of agreement define bias +1.96 ∗ CV. CL: central laboratory; CV: coefficient of variation; HC201+: HemoCue 201+; HC201RT: HemoCue 201 RT; MARD: mean absolute relative difference.

## Data Availability

The data used to support the findings of this study are available from the corresponding author upon request.

## Abbreviations

BMI: Body mass index
CDC4G: Changing Diagnostic Criteria for the Diagnosis of Gestational Diabetes
CL: Central laboratory
EQA: External quality assessment
GDM: Gestational diabetes mellitus
HAPO: Hyperglycemia and Adverse Pregnancy Outcomes
HC201+: HemoCue Glucose 201+
HC201RT: HemoCue Glucose 201 RT
ID GC-MS: Isotope dilution gas chromatography-mass spectrometry
ISO: International Organization for Standardization
OGTT: Oral glucose tolerance test
POC: Point of care
SNBHW: Swedish National Board of Health and Welfare
WHO: World Health Organization.
